# No more ‘business as usual’ with audit and feedback interventions: towards an agenda for a reinvigorated intervention

**DOI:** 10.1186/1748-5908-9-14

**Published:** 2014-01-17

**Authors:** Noah M Ivers, Anne Sales, Heather Colquhoun, Susan Michie, Robbie Foy, Jill J Francis, Jeremy M Grimshaw

**Affiliations:** 1 Department of Family and Community Medicine, Institute for Health Systems Solutions and Virtual Care, Women’s College Hospital, University of Toronto, 77 Grenville Street, Toronto, ON M5S 1B3, Canada; 2 Center for Clinical Management Research, VA Ann Arbor Healthcare System, University of Michigan, 2215 Fuller Road, Mail Stop 152, Ann Arbor, MI 48105, USA; 3 Ottawa Hospital Research Institute, Clinical Epidemiology Program, The Ottawa Hospital, General Campus, 501 Smyth Road, Centre for Practice Changing Research, Box 201B, Ottawa, ON K1H 8L6, Canada; 4 Centre for Outcomes Research and Effectiveness, Department of Clinical, Educational and Health Psychology University College London, 1-19 Torrington Place, London, WC1E 7HB, UK; 5 Leeds Institute of Health Sciences, University of Leeds, Charles Thackrah Building, 101 Clarendon Road, Leeds LS2 9LJ, UK; 6 School of Health Sciences, City University London, Northampton Square, London EC1V 0HB, UK; 7 Clinical Epidemiology Program, Ottawa Health Research Institute, University of Ottawa, 1053 Carling Avenue, Administration Building, Room 2-017, Ottawa, ON K1Y 4E9, Canada

**Keywords:** Audit and feedback, Synthesis, Best practice, Implementation, Optimization

## Abstract

**Background:**

Audit and feedback interventions in healthcare have been found to be effective, but there has been little progress with respect to understanding their mechanisms of action or identifying their key ‘active ingredients.’

**Discussion:**

Given the increasing use of audit and feedback to improve quality of care, it is imperative to focus further research on understanding how and when it works best. In this paper, we argue that continuing the ‘business as usual’ approach to evaluating two-arm trials of audit and feedback interventions against usual care for common problems and settings is unlikely to contribute new generalizable findings. Future audit and feedback trials should incorporate evidence- and theory-based best practices, and address known gaps in the literature.

**Summary:**

We offer an agenda for high-priority research topics for implementation researchers that focuses on reviewing best practices for designing audit and feedback interventions to optimize effectiveness.

## Background

Audit and feedback (A&F) involves providing a recipient with a summary of their performance over a specified period of time and is a common strategy to promote the implementation of evidence-based practices. A&F is used widely in healthcare by a range of stakeholders, including research funders and health system payers, delivery organizations, professional groups and researchers, to monitor and change health professionals’ behaviour, both to increase accountability and to improve quality of care. A&F is an improvement over self-assessment [1] or self-monitoring [2] as it can provide objective data regarding discrepancies between current practice and target performance, as well as comparisons of performance to other health professionals. The recognition of suboptimal performance can act as a cue for action, encouraging those who are both motivated and capable to take action to reduce the discrepancy.

The effectiveness of A&F has been evaluated in the third update of a Cochrane review, which included 140 randomized trials of A&F conducted across many clinical conditions and settings around the world. The review found that A&F leads to a median 4.3% absolute improvement (interquartile range 0.5% to 16%) in provider compliance with desired practice [3]. One-quarter of A&F interventions had a relatively large, positive effect on quality of care, while another quarter had a negative or null effect. The challenge of identifying factors that differentiate more and less successful A&F interventions is exacerbated by poor reporting of both intervention components and contextual factors in the literature [4]. Furthermore, most A&F interventions tested in RCTs are designed without explicitly building on previous research or extant theory [5,6]. As a result, there has been little progress with respect to identifying the key ingredients for a successful A&F intervention or understanding the mechanisms of action of effective A&F interventions in healthcare [7]. Given the now established effectiveness of A&F, its popularity as an intervention is likely to continue; it is imperative that we improve our understanding of how, when and why it achieves large effects.

A&F has been used as a method for changing behaviour in educational [8], organizational [9-11], cognitive [12], and health psychology [13,14]; research in these areas has produced hypotheses about mechanisms of action. The effectiveness of A&F may be influenced by the characteristics of the targeted behavior, the recipients, their context, and the components of the A&F intervention itself [9,15,16]. To optimize the effectiveness of A&F, we need to understand how to account for these characteristics to design better interventions. The most recent Cochrane review advanced knowledge about A&F by using theory underpinning A&F to guide its evidence synthesis. For example, it incorporated re-analyses [14,17] of the previous Cochrane review informed by control theory [13] and feedback intervention theory [10], resulting in the finding that feedback is more effective when accompanied by both explicit goals and an action plan. This provides, for the first time, clear advice as to key components that should be incorporated. While this theory-based synthesis made good progress, we need to build on this to identify other key components that will allow practitioners and policy-makers to adopt or adapt A&F with sufficient confidence of achieving high impacts on service delivery and outcomes.

Just as the need to identify research gaps and prioritize research agendas is accepted for clinical research [18], it is also a prerequisite for making substantial progress in implementation research [19]. Rather than continuing with ‘business as usual,’ we believe that a coordinated effort and a systematic approach are now required to determine how best to design and deliver A&F. To this end, a group of international experts from a range of disciplinary backgrounds met for two days in December, 2012. The objectives were: to develop guidance based on current best practices for those developing A&F interventions; and to identify high-priority research topics and opportunities for optimizing A&F effectiveness. Participants were purposively sought based on their contributions to A&F or related literature in healthcare or their roles as potential knowledge users. The list of participants and agenda of the meeting are provided in the (see Additional file 1). This paper summarizes the outcomes of that meeting.

## Discussion

### Objective 1. Guidance for developing A&F interventions

In the process of developing guidance for A&F intervention development, three linked concepts were identified: identifying known best practices; applying relevant theory when operationalizing best practices; and considering intervention components as factors to be manipulated.

### 1a. Best practices in design of A&F interventions

Although the ‘ideal’ design for A&F interventions depends on the recipient, their context, and the targeted behaviour, ‘best practices’ can be guided by theory and evidence. The consensus judgment of ‘best practices’ for designing A&F interventions are summarized in Table 1, and evidence in support of these practices is reviewed below.

**Table 1:** Tentative ‘best practices’ when designing A&F interventions

Audit components	Data are valid
	Data is based on recent performance
	Data are about the individual/team’s own behavior(s)
	Audit cycles are repeated, with new data presented over time
Feedback components	Presentation is multi-modal including either text and talking or text and graphical materials
	Delivery comes from a trusted source
	Feedback includes comparison data with relevant others
Nature of the behaviour change required	Targeted behavior is likely to be amenable to feedback
	Recipients are capable and responsible for improvement
Targets, goals, and action plan	The target performance is provided
	Goals set for the target behaviour are aligned with personal and organizational priorities
	Goals for target behaviour are specific, measurable, achievable, relevant, time-bound
	A clear action plan is provided when discrepancies are evident

### 1a.i. A&F components

The meta-regression in the Cochrane review of A&F indicated that feedback is more effective when it is presented both verbally and in writing than when using only one modality and when the source (*i.e*., person delivering the feedback) is a supervisor or senior colleague rather than unknown investigators. Conversely, feedback is less effective when it comes from a regulatory body, agreeing with qualitative work suggesting that feedback perceived to be potentially punitive appeared less effective than a supportive approach [15,20]. Feedback intervention theory [10] suggests that feedback that captures the recipient’s attention and directs it toward a specific behaviour is more likely to be successful, but when A&F comes from a regulatory body, the recipient may be more likely to activate affective processes (*e.g*., distress) which distract attention from the specific task requiring change. Diffusion of innovation theory [21] would suggest that feedback from opinion leaders is likely to be more effective, but this was not investigated by the Cochrane reviews of opinion leaders [22] or the A&F review. Aside from exerting social pressure, in-person delivery of feedback could allow for tailoring of the message according to recipient characteristics. This type of process appears to offer a potentially important and readily testable hypothesis. Finally, the A&F review suggested that repeated feedback cycles led to greater improvements in performance than once-only feedback. In part, this may be because recipients are more likely to perceive the data as relevant and accurate when it is delivered closer in time to their own performance [9].

Although the Cochrane review of A&F did not make a specific recommendation, there is a theoretical basis for including comparison data in feedback as a means of motivating recipients to change behavior. Specifically, the theory of planned behaviour emphasizes the potential for social pressure to influence intentions to change professional practice [23]; this could help explain why feedback delivered by senior colleagues tends to be more effective than feedback delivered by unknown investigators. When feedback is delivered via written reports, a preferred approach to the inclusion of normative data for comparison has been identified through a large head-to-head trial [24]. In particular, recipients who received feedback with ‘achievable benchmarks of care’ [25] (*i.e*., comparing to the top 10% of peers) had greater improvements in processes of care than those receiving feedback where the median performance of peers was highlighted.

### 1a.ii. Nature of the behaviour change required

The Cochrane review indicated that feedback is most effective when targeting performance with large room for improvement. This may be attributed to larger discrepancy with the comparator, resulting in greater intention to take action, or may reflect the absence of ceiling effects, or both. In addition, the likelihood of feedback identifying a discrepancy between actual and ideal care leading to changed behaviour may depend upon the degree to which recipients believe they have control over that behavior and are committed to change [11]. Feedback should describe achievable behaviors that are ideally desired by the recipient and implementable by the member(s) of the healthcare team responsible for enacting the desired change [15]. This may, in part, explain the tentative findings in the Cochrane review that A&F was less effective when the targeted behaviour changes were more complex (*e.g*., when special skills are required to implement the desired change).

An observational study showed that clinical practice recommendations rated as incompatible with clinician values and norms were associated with lower compliance at baseline but greater behavior change following feedback than recommendations perceived as highly compatible [26]. This illustrates the potential for A&F to have an effect even upon targeted behaviours reported by clinicians not to be compatible with their values [20,24].

### 1a.iii. Targets, goals, and action planning

The Cochrane review findings that feedback interventions were more effective when they included explicit goals and an action plan are not surprising [3]. This is consistent with theories [11,13] positing that goals can make feedback more salient and action plans specify and facilitate the steps required to achieve goals. When goals are more specific, this process is more effective [9]; ideal goals are commonly considered to be specific, measurable, achievable, relevant and time-bound [27]. Feedback intervention theory proposes that action plans help focus recipients’ attention more productively on the task [10]. In healthcare, providers often manage multiple competing goals with their patients [28]; when A&F successfully directs attention toward specific tasks, it may influence prioritization of these goals.

From a cognitive perspective, receipt of feedback that indicates a discrepancy between expected and actual performance may generate a response described as cognitive dissonance [12]. In this case, participants may attempt to reduce the discrepancy by planning to improve [13] or by undermining the saliency of the discrepancy by discounting the data (or the goals targeted) as invalid [16]. Facilitating the former and reducing the latter is an important aspect of designing effective A&F interventions.

### 1b. Applying relevant theory to improve design and increase contribution to the literature

While the optimal design of A&F will vary to some extent for each purpose or setting, the strategies chosen for each component (Table 1) should be justified both by empirical evidence and theory [29]. If theory is used, it allows the evaluation of mechanisms of action, thereby contributing to an understanding of why any observed effects occurred. Although a single, all-encompassing, highly predictive theory of A&F is unlikely in the foreseeable future, a number of theories and theoretical constructs are informative [12]. For example, at the individual level, increasing self-efficacy [30-32] and goal commitment [11,33] is likely to increase performance. Organizational and system-level factors that may affect recipients’ ability to act upon a discrepancy highlighted by feedback could also be considered, including competing priorities for the recipients, teams and organizations [34]. This has implications not only with respect to potential contextual effect modifiers, but for the design of the intervention itself: the ideal number and nature of goal-directed behaviours targeted by the feedback may vary by setting and by the organizational level at which the feedback is directed. For example, managers may be able to assimilate and act on a greater number of topics than frontline clinicians, but may lack direct control over specific behaviors.

### 1c. Manipulating levers: applying best practices within real-world constraints

A&F interventions consist of multiple components, each requiring attention during the design stage; ensuring the inclusion of all desirable components and designing each component in the optimal fashion is rarely possible. We recommend considering individual intervention components as ‘levers’ to be manipulated when working within setting-specific or study design constraints. For example, if circumstances dictate that the delivery of feedback cannot be repeated in a reasonable timeframe, extra attention should be paid to other aspects of the intervention to ensure that discrepancies between actual and desired practice are salient and actionable.

In addition, co-interventions, tailored to overcome identified barriers and boost facilitators, may help if feedback alone seems unlikely to activate the desired response [35]. Ideally, co-interventions that are likely to enhance the process and outcome of A&F interventions should be selected, based on careful consideration of factors that are likely to influence the target behaviours. This gives the opportunity to investigate potential synergy between multiple implementation strategies. For example, while it may seem intuitive to combine payment incentives with A&F, in recipients who are already highly motivated to achieve the goals but have limited perceived control over the outcomes, this combination may not be effective [33]. Alternatively, co-interventions that increase recipients’ skills in quality improvement may be useful because skill development and resultant self-efficacy may increase the likelihood that feedback will lead to improved performance [11,36]. Since multifaceted interventions featuring A&F may offer little benefit over A&F alone [3], cost-effectiveness of co-interventions should also be considered.

### Objective 1 summary – best practices for implementing effective A&F interventions

To summarize our guidance for developing A&F interventions, the best practices outlined in Table 1 should be considered and applied when possible. In particular, prior to conducting A&F, the nature of the behaviour change required to improve performance should be elucidated in order to develop clear action plans. Theory and evidence should be made explicit with respect to proposed design choices when manipulating the design of individual intervention components, and the expected mechanisms of action should be justified to aid interpretation.

### Objective 2. High-priority research topics and opportunities for optimizing audit and feedback

There is a need for future research to turn its focus from evaluating A&F interventions against usual care to explicitly evaluating different types of A&F. In particular, confirmatory head-to-head trials examining different strategies for implementing the tentative recommendations in Table 1 would provide a valuable contribution to knowledge and practice. However, even if the components featured in Table 1 were optimized, additional components of A&F (that might be currently known or unknown) or co-interventions would be worthy of testing. Additionally, further knowledge regarding potential effect modifiers is needed.

Examples of potentially fruitful research topics that could lead to enhancing the effectiveness of A&F are listed in Table 2. These topics are organized by factors related to: a) contextual and recipient characteristics that may moderate A&F effectiveness, b) the design of the A&F intervention itself, and c) the characteristics of the targeted behaviour change. Knowledge about organizational resources and the nature of the team responsible for achieving the outcome of interest could help explain variability in the effects of A&F interventions [37]. The complexity of clinical practice recommendations and the degree to which benefits can readily be observed may play an important role [21,38-40]. More information is needed to understand whether some behaviours are more amenable to change through A&F and/or how to adapt the design of A&F accordingly.

**Table 2:** Example research topics for optimizing audit and feedback

Factors related to context and/or recipient	
Characteristics of the recipient	• Engagement in audit and/or in feedback design
	• Goal orientation of recipients
	• Degree of motivation to improve performance
	• Training of recipients to understand and act on feedback
	• Profession of recipient and/or multi-disciplinary feedback
Characteristics of the setting	• Location (*e.g*., hospital versus clinic, national setting)
	• Organizational resources
	• Size of the team responsible for outcomes of interest
Co-interventions	• Time and/or standardized support to reflect upon feedback
	• Impact of combining A&F with one of the following:
	• Incentives or penalties (financial, CME, licensing)
	• Tools and practise aids (clinical decision tool)
	• Education (academic detailing, group learning)
	• Practice redesign (coaches, facilitation, mentorship)
Factors related to intervention design	
Nature of delivery of the information	• Mode of delivery of feedback (*e.g*., paper, electronic, face-to-face)
	• Length, duration
	• Perceived credibility of the source and/or competence of the presenter
	• Different sources (peer versus supervisor versus external group)
	• Frequency of feedback
	• Role of social pressure, dissemination/visibility of information to peer-group
Nature of the content	• Sign of the message (positive versus negative)
	• Graded feedback (starting positive)
	• Type of benchmarks and/or comparison information
	• Type action plans or correct solution information
	• Level of aggregation of feedback data (individual versus team)
	• Role for intermediate outcomes/process measures versus patient-level outcomes
Characteristics of the targeted behaviours	• Perceived importance of the target relative to other priorities
	• Observability of improvement (whether impact of using a new practice can be seen quickly)
	• The degree to which the recommended practice requires changes in habits and routines
	• Complexity of targeted behaviours (number of indicators reported or behavioural changes required and skill level necessary for desired behaviour change)

There are opportunities to embed research within ongoing organizational initiatives at low cost in settings where routine data are available and where mechanisms for audit are in place, and this should be considered in the planning and conduct of A&F studies [41]. In particular, A&F researchers could partner with those providing routine feedback to healthcare providers to test variations of intervention components that might increase effectiveness. Administrators of quality improvement initiatives involving A&F may be keen to participate in head-to-head trials comparing different A&F designs because, in this case, all providers receive an intervention, while management learns about how their programs may be improved. Thus, settings where valid data for audit and mechanisms for feedback are already established are potential implementation research laboratories, enabling relatively rapid testing of multiple intervention components.

There is evidence suggesting that physicians respond differently than other health professionals to implementation interventions [42], and it is possible that this is also true for A&F. Even within the same broad professional discipline, there may be differential responses related to values and contexts of different groupings (*e.g*., primary and secondary care providers). The Cochrane review found little difference in effect size between A&F targeting physicians and non-physicians, but 86% of the trials focused on physicians. It is plausible that the effectiveness of A&F and key effect modifiers may vary in less-studied settings where non-physicians are the primary providers, such as home care or long-term care.

Opportunities for advancing the science related to A&F may be found through consideration of different study designs. When evaluating the addition of a single component, such as adding action plans to feedback, a two-arm, head-to-head trial is a logical option [43]. However, where there is an opportunity to consider many A&F-related factors at once, a multi-phase optimization strategy including a fractional factorial trial offers an efficient approach [44]. In such a study design, an investigator could simultaneously test various components of A&F content and delivery of the intervention. Existing A&F-relevant theories should be used to select the intervention components to be studied and to generate hypotheses regarding the interaction between such components and contextual characteristics, including those of the recipient and of the targeted behaviour. In addition, a process evaluation within a trial can be used to investigate and develop program-specific theory [45]. Examining mechanisms of action and qualitative studies prior to trials may help identify barriers and facilitators to change that can inform intervention design [15].

### Objective 2 summary – opportunities to improve knowledge regarding how to optimize A&F

To summarize, we believe that future A&F research should prioritize studies that aim to determine how the effect of the intervention may be optimized rather than assessing whether the intervention can increase the implementation of evidence-based practices. This justification is similar to the arguments made in support of the shift toward comparative effectiveness research elsewhere in healthcare. Table 2 provides example topics in which greater understanding is needed to determine how best to utilize A&F. On the basis of the considerations reviewed above, we recommend that, prior to directing further research effort toward new trials of A&F versus usual care, investigators consider the following questions:

1. How could a proposed study add to present empirical knowledge? For example, is there a good justification for considering a particular intervention component and a clear hypothesis regarding how it will alter the effectiveness of A&F?

2. To what degree can the components of the A&F intervention be sufficiently defined and operationalized across different settings? For example, how costly is the intervention to replicate, and is there potential for sustainability and spread?

3. Has some pilot work been conducted? For example, is the feedback interpreted as intended, and has the design been iteratively optimized prior to scaling up?

## Summary

A&F has the potential to significantly improve quality of care, but despite more than 140 published RCTs, important knowledge gaps remain regarding when it will work best and why, and how to design reliable, effective A&F interventions across settings and provider groups. The lack of systematic, coordinated research in this area, as well as gaps in study reporting, contribute to this problem. To determine changes required to the ‘business as usual’ approach focusing on trials of A&F versus usual care, we gathered a multidisciplinary group of international researchers and knowledge users to develop an agenda for future research in the field. Participants recognized the tension between research and action and urged the research community to capitalize on opportunities to simultaneously contribute to implementation science while conducting implementation. The discussions we summarize here were driven by the findings from the most recent Cochrane review of A&F interventions in healthcare, and the collective experiences and research programs of the meeting participants. The limitations of the recommendations described here reflect the limitations of the studies reviewed. In addition, our considerations only reflect opinions expressed at the meeting. We deliberately sought to invite a wide range of individuals to the meeting to maximize diversity, but with a maximum of 30 attendees, there may have been important perspectives omitted. We acknowledge that there is no present universally accepted, comprehensive list of A&F intervention components.

We have summarized best practice recommendations for the design of A&F based on currently available evidence and have proposed examples of high priority research questions that could move the field forward. Future A&F interventions should feature well-described and carefully justified components, with the theoretical rationale made explicit. Rather than comparisons of A&F versus usual care, head-to-head comparisons of interventions with embedded process evaluations are encouraged to determine how to optimize effect size. Researchers, funders and other stakeholders should assess new A&F studies critically to evaluate the extent to which they contribute new knowledge to the field, and endeavor to draw on best evidence when designing and implementing interventions.

## Competing interests

The authors declare they have no competing interests.

## Authors’ contributions

NMI and JMG conceived the paper. NMI, AS, and HC co-wrote the first draft. All authors provided critical intellectual input and approved the final version.

## Supplementary Material

Additional file 1 Summary of meeting agenda and participant characteristics. Click here for file
