# Supplementary material for: No more ‘business as usual’ with audit and feedback interventions: towards an agenda for a reinvigorated intervention
Source: Implement Sci. 2014 Jan 17;9:14. doi: 10.1186/1748-5908-9-14 (PMC3896824; doi:10.1186/1748-5908-9-14)
Supplement: Additional file 1 — Summary of meeting agenda and participant characteristics. [file 1748-5908-9-14-S1.docx]

**SUPPLEMENTARY FILE 1**

We received letters of support and interest from 24 experts across 8 countries; the agenda is reviewed below and the disciplines, interests and contextual backgrounds of the participants are summarized in Supplementary Table 1. Further details regarding the participants and the agenda of the meeting are available online at: **http://whatiskt.wikispaces.com/Audit+and+Feedback+Meeting**.

The two-day meeting discussed the following:

1. The findings from the Cochrane review of A&F and the results of the cumulative meta-analysis showing stable effect sizes over 25 years. The findings from a secondary analysis of the Cochrane review that a wide range of A&F intervention components were used without explicit justification by investigators in the published trial reports.

2. Theory-informed intervention design and the need for a program-specific theory of A&F to guide the selection of intervention components.

3. Methodological options for investigating optimal intervention design were considered, including iterative approaches to improve design and fractional factorial trials as potential methods to determine ideal intervention components (as in Collins et al. Ann Behav Med. 2011 Apr;41(2):208-26. doi: 10.1007/s12160-010-9253-x.).

Small groups, using a modified nominal group technique, focused on intrinsic factors (intervention components), extrinsic factors (context), or mechanism of action to consider (i) a wide range of potential A&F components and effect modifiers, and (ii) how to prioritize questions related to such factors for future research. Deliberation results from small groups were disseminated amongst the whole group to ensure accuracy and to elicit further input.

The academic discussion was complemented by participants describing their experience developing and delivering feedback to health care providers. These ‘real-world’ examples highlighted the practical feasibility constraints and issues that must be considered when delivering A&F. They also raised awareness of the opportunities to leverage ‘natural laboratories’ for A&F research.

**Supplementary Table 1. Participant characteristics**

| **Name** | **Country** | **Expertise** |
| --- | --- | --- |
| *Noah Ivers | Canada | Lead author, Cochrane review of A&F |
| *Jeremy Grimshaw | Canada | CRC in Knowledge Transfer and Uptake |
| *Heather Colquhoun | Canada | Behavioural theory and A&F |
| Jamie Brehaut | Canada | Cognitive psychology |
| Janet Curran | Canada | Tailoring intervention design based on barriers |
| Merrick Zwarenstein | Canada | Pragmatic health services trials |
| Mark Chignall | Canada | Human factors engineering for intervention design |
| Sumit Majumdar | Canada | Quality improvement trials |
| ^Craig Campbell | Canada | CPD for Royal College of Physicians |
| ^Lynn Dionne | Canada | Health Quality Ontario quality improvement plans |
| ^Donna Angus | Canada | Director of KT, Alberta Innovates-Health Solutions |
| ^George Collier | Canada | Manager, Nova Scotia Health Research Foundation |
| ^Tupper Bean | Canada | Primary care quality improvement consulting |
| *Susan Michie | UK | Health psychology |
| *Jill Francis | UK | Health psychology |
| Robbie Foy | UK | Audit and feedback trials |
| Richard Baker | UK | Implementation of evidence |
| Mary Dixon Woods | UK | Medical sociology |
| *Anne Sales | USA | A&F for nursing and long term care, editor Implementation Science |
| Michael Halasy | USA | Organizational theory and A&F |
| Sylvia Hysong | USA | Industrial psychology and A&F |
| ^Steve Ornstein | USA | Quality improvement trials, founder of PPRNet |
| ^Cara Litvin | USA | Quality improvement involving A&F |
| ^David Price | USA | CPD and Quality improvement at Kaiser |
| Gro Jamtvedt | Norway | Author of Cochrane A&F review, Physiotherapy |
| Signe Flottorp | Norway | Author of Cochrane A&F review, Family medicine |
| Denise O’Connor | Australia | Health professional behavior change interventions |
| Simon French | Australia | Author of Cochrane A&F review, Chiropractic |
| Sue Wells | NZ | Implementation of web-based decision support |
| Michel Wensing | Holland | Primary care research, editor Implementation Science |

* = Organizing committee, ^ = Knowledge users
